# Preoperative Evaluation of Parkinson’s Disease

**DOI:** 10.7759/cureus.15933

**Published:** 2021-06-26

**Authors:** Bianca C Woodruff, David Garcia, Shobhana Chaudhari

**Affiliations:** 1 Anesthesiology, Metropolitan Hospital Center, New York, USA; 2 Geriatrics, Metropolitan Hospital Center, New York, USA

**Keywords:** preoperative evaluation, geriatric patient, parkinson’s disease, multi-morbidity, polypharmacy

## Abstract

Parkinson’s disease (PD) is a common disease of older adults. It presents a unique set of anesthetic challenges. With increasing life expectancy and the rapid growth of oldest-old patients, it is now more likely for anesthesiologists to encounter older patients with PD. Due to polypharmacy in the older adults with PD a potential for drug interactions during and after surgery needs to be considered. More data from prospective, multicenter trials about short- and long-term outcomes of anesthesia in PD patients are needed.

## Introduction

Parkinson’s disease (PD) is one of the common neurological diseases in older adults. It affects approximately one million individuals in the United States and 10 million individuals worldwide [1]. It is a progressive neurodegenerative disease of the central nervous system caused by the loss of dopaminergic neurons in the basal ganglia of the brain and is characterized by a classical triad of resting tremor, muscle rigidity, and bradykinesia [2]. PD is an important cause of perioperative morbidity whereby complications commonly arise from the impact of the disease on the respiratory, cardiovascular, gastrointestinal, urological, and neurological systems. The rates of postoperative aspiration pneumonia (due to laryngeal/pharyngeal muscle dyskinesia), post-extubation laryngospasm, orthostatic dysregulation, arrhythmias, bacterial infections, urinary tract infections, sialorrhea, gastroesophageal reflux disease (GERD), gastroparesis with chronic constipation, falls, prolonged hospital stay, postoperative hallucinations and delirium, cognitive impairments, and greater need for post-hospitalization rehabilitation are significantly increased in this population [3]. Polypharmacy in older PD patients can lead to potential interactions with anesthetic drugs. Here we present a challenging case of an older adult with PD who needed the preoperative evaluation. 

## Case presentation

An 80-year-old female with a medical history of PD, history of multiple intracranial aneurysms with multiple embolizations secondary to subarachnoid bleed complicated by left carotid-cavernous fistula formation, hypertension, systolic chronic heart failure with reduced ejection fraction, chronic kidney disease stage 3, and left bundle branch block presented at the geriatric clinic for a preoperative evaluation. An elective cerebral angiography and possible transvenous embolization of the left carotid-cavernous fistula was planned to treat the patient’s decreasing vision, diplopia, and headache. The past surgical history was significant for bilateral cataract extraction, cholecystectomy, and multiple cerebral angiograms with embolization of the right ophthalmic artery aneurysm.

Home medication included three medications for PD as selegiline 5 mg BID, rotigotine transdermal patch 2 mg/24 h, and trihexyphenidil 2 mg TID due to left-hand tremors and rigidity progression. Additionally, she was on fosinopril 40 mg, calcium carbonate + Vitamin D 600/400 mg BID, Vitamin B12 1 mg, acetaminophen pro re nata (PRN) 500 mg, aspirin 81 mg, and clopidogrel 75 mg. The patient was ambulatory without an assistive device, with trouble initiating movement, a typical shuffling gait, and reported an exercise tolerance of more than 15 blocks.

On physical examination, she was awake, alert, and oriented x 3 and had “pill rolling” tremor and cogwheel rigidity of the left upper extremity. Her vital signs were blood pressure (BP) 142/64, heart rate (HR) 93 beats/min, respiratory rate (RR) 18 breaths/min, afebrile, and O2 saturation 98% on room air. The remaining physical review was unremarkable. Her laboratory examination showed chronic normocytic/normochromic anemia with a normal anemia workup. Her electrocardiogram was significant for a known left bundle branch block. Her last echocardiogram confirmed a left ventricular ejection fraction of 40%-45%. 

The revised cardiac risk index for the patient was low to intermediate for a high-risk procedure. The patient was advised to hold selegiline on the day of the procedure due to possible interactions with intra-operative medications and the elevated risk of precipitating serotonin syndrome. She was recommended to take all other medications early in the morning and to resume her anti-Parkinson medication as soon as possible after the procedure. The patient underwent the proposed procedure under general anesthesia, was extubated in the operating room, and transferred to the ICU for close monitoring. The procedure was a successful surpass streamline flow diverter insertion of a complex one centimeter left cavernous segment aneurysm, as well as a three-millimeter, left ophthalmic artery aneurysm along a dysmorphic segment. The surpass streamline flow diverter is a cobalt chromium and platinum tungsten braided, self-expandable tube. The procedure was complicated by rupture of the cavernous aneurysm with cavernous carotid fistula draining towards the pterygoid veins, which was treated with placement of a second surpass streamline flow diverter. During the postoperative management, the patient maintained her baseline physical exam and mental status but presented with left eye pain and mild hypertension that was treated successfully with dexamethasone and labetalol. The PD medications were restarted during the ICU stay, without any adverse effects. On a postoperative day five, the patient was discharged from the hospital with dexamethasone tapering, aspirin 325 mg, clopidogrel 75 mg, and close follow-up visits with the Geriatrics and Neurology department.

## Discussion

Antiparkinsonian medications should not be withheld. A missed dose can lead to increased rigidity, loss of balance, agitation, and confusion. If PD medication is withheld for too long, neuroleptic malignant syndrome or parkinsonism-hyperpyrexia syndrome can develop. Thus, to avoid symptom exacerbation and other adverse effects the usual drug regimen should be continued until just before the induction of anesthesia, except in patients undergoing surgery for deep brain stimulators [4]. In that case, medications should be withheld in the morning only. Levodopa’s half-life is only one to three hours. Enteral administration is preferred due to the fact that IV administration of levodopa increases the risk of interactions with anesthetic agents and can cause arrhythmias and hypertension via its metabolite, dopamine. If postoperative enteral administration is impossible, a transdermal application of rotigotin should be used. 

Furthermore, PD patients should be the first surgery cases of the day [5]. Placing these patients first, and ensuring they stay on their scheduled PD medications during their nil per os (NPO) status, is important. If scheduling as the first case is not possible, PD patients should be allowed to take their PD medications with a sip of water during the day of surgery. 

Patients on levodopa treatment can have severe nausea and vomiting, intensified by opioids given during general anesthesia. They are prone to dehydration and hypovolemia, can develop orthostatic hypotension and/or arrhythmia, and are susceptible to postoperative delirium with visual hallucinations, which can also be intensified by intraoperative opioids and benzodiazepines. Adequate fluid management during the perioperative period is crucial to prevent orthostatic hypotension.

Depression is a common neuropsychiatric manifestation of PD, and selective serotonin reuptake inhibitors (SSRI) medications prescribed for many patients with PD to treat depression can interact with serotonergic drugs given during the perioperative period (like tramadol, meperidine, fentanyl, ondansetron, and granisetron). Such drug-drug interaction increases the risk for potentially fatal serotonin syndrome and should be avoided. 

Acetylcholinesterase inhibitors (rivastigmine, donepezil, and galantamine), commonly prescribed to treat PD dementia, have been associated with a prolonged effect of succinylcholine (up to 50 min) and increased resistance to non-depolarizing neuromuscular blocking drugs.

For evaluation of the anesthetic plan, the following points have to be considered: thiopental decreases the release of dopamine in the striatal synaptosome. One case of peri- and postoperative rigor with parkinsonian episodes seen after induction with 375 mg thiopental has been described [6]. Etomidate can cause myoclonus that can be decreased with the simultaneous use of benzodiazepines [3]. Ketamine can produce intraoperative unprecedented BP responses and postoperative hallucinations and is, therefore, theoretically contraindicated. However, it can also provide patient comfort and resolution of dyskinesia via its N-methyl-D-aspartate (NMDA) antagonist properties. At low doses (0.1-0.5 mg/kg IV) ketamine is a safe and useful temporary adjunct to long-term treatment when dopamine medications are missed within the perioperative setting [7]. Propofol, the most commonly used anesthetic drug, has been reported to induce dyskinesia. Propofol increases striatal levels of gamma-aminobutyric acid (GABA), and increased concentrations of GABA receptors have been observed postmortem in the globus pallidus internus of parkinsonian patients prone to dyskinesia, indicating that these patients may be more sensitive to the effects of GABA than patients without PD [8]. Research has found that propofol inhibits neuronal activity in the subthalamic nucleus, and that lesion of the subthalami nucleus can result in disorders of excessive movement such as ballism and chorea. Nonetheless, propofol is often used in patients with PD undergoing surgery because of its favorable anesthetic and antiemetic properties. However, clinicians should remain aware of the increased risk for propofol-induced dyskinesia in this group, especially in those with advanced disease. As described in a report published in 2006, dexmedetomidine was found to manage propofol-induced dyskinesia successfully in a patient with PD undergoing deep brain stimulator placement [9]. Regional anesthesia (RA) might be preferred due to its advantages: does not mask a PD exacerbation, enables intraoperative monitoring of parkinsonian symptoms, allows earlier return to postoperative oral intake, reduces the use of systemic opioids, does not require anticholinergic reversal agents, and has a lower risk of aspiration. Low-dose midazolam in combination with low-dose remifentanil infusion could be used. However, the disadvantages of RA in PD patients must also be considered. The PD tremor can interfere not only with the monitoring device but might also make surgery impossible. Available clinical evidence does not show persisting cognitive decline in PD patients after repeated general anesthesia [10]. Inhalational anesthetics, which have a complex impact on the brain, can lead to an increase of extracellular dopamine via inhibition of synaptic reuptake and increased release of dopamine [11-12]. Neuromuscular blocking drugs are generally safe to use in PD. The ideal agent is rocuronium because of its antidote sugammadex. Neostigmine should be used with caution due to its thickening effect on airway secretions. Opioids are safe in PD except for meperidine and tramadol due to the increased risk of precipitating serotonin syndrome. Some cases of increased muscle rigidity in PD patients given high opioid doses have been published. Sialorrhea can complicate airway management but this can be reduced with glycopyrrolate. Due to the increased prevalence of gastroparesis and GERD in PD patients, a rapid sequence induction is preferable. Centrally acting anticholinergics (atropine) can precipitate a central anticholinergic syndrome with confusion, somnolence, and restlessness. Glycopyrrolate is a safe peripherally acting alternative. Antimuscarinic drugs like neostigmine increase the viscosity of saliva and impair swallowing, thereby, increasing the risk of postoperative bronchospasm in patients with PD who have obstructive dysfunction due to parasympathetic overactivity. For postoperative nausea and vomiting, prophylaxis and treatment should be approached with caution. Consider total IV anesthesia agents, such as propofol and dexmedetomidine, which have opioid-sparing effects. Dexamethasone and serotonin antagonists are good agents for prophylaxis. Phenothiazines should be avoided because they may lead to Parkinson's exacerbation.

Oral PD medication should be restarted after surgery as soon as possible, best case in the evening of the day of surgery, as severe akinesia, can have many negative perioperative effects. Levodopa can be administered via nasogastric (NG) tube. Rectal application is not recommended due to levodopa’s resorption in the proximal jejunum [13]. In case the enteral application is not possible, alternative treatments can be used: amantadine sulfate (200 mg IV once daily), apomorphine [1-2 mg subcutaneous (SC), half life is only 30 min, continuous SC application recommended], and rotigotine (transdermal patch 4 mg/24 h) [14]. Early rehabilitation with early postoperative mobilization is strongly recommended. 

Postoperative delirium is particularly common and challenging in PD. With rates as high as 60%, postoperative delirium is best managed by non-pharmacological methods like reorientation (talking to the patient and explaining time, location, and your role) and providing a suitable care environment [15]. Drugs that precipitate or exacerbate PD like metoclopramide, phenothiazine (promethazine, prochlorperazine), butyrophenone (haloperidol, droperidol), or atypical anti-psychotics should be withheld prior to surgery (Table 1). Furthermore, opioids such as meperidine or tramadol should be discontinued prior to their procedure due to their interaction with PD medications (like monoamine oxidase B inhibitor, selegiline) and their risk of precipitating a serotonin syndrome.

**Table 1:** Drugs that precipitate or exacerbate PD. PD, Parkinson's disease

Drugs that precipitate or exacerbate PD	
Antiemetics	Metoclopramide
Typical antipsychotics	Chlorpromazine, prochlorperazine, perphenazine, fluphenazine, promethazine, haloperidol, droperidol, pimozide, sulpiride
Atypical antipsychotics	Risperidone, olanzapine, ziprasidone, aripiprazole

## Conclusions

Polypharmacy, in older PD patients, can lead to potential drug interactions with anesthetic drugs and techniques. This article highlights the importance of preoperative evaluation of an older PD patient. Unfortunately, much of the evidence about the safety of various anesthetic drugs or techniques used in PD is based on single case reports or small case series. More data from prospective, multicenter trials about the short- and long-term consequences of general and regional anesthesia in older patients with PD are needed.
